# A qualitative study of older informal carers’ experiences and perceptions of their caring role

**DOI:** 10.1016/j.maturitas.2019.03.006

**Published:** 2019-06

**Authors:** Nan Greenwood, Carole Pound, Sally Brearley, Raymond Smith

**Affiliations:** a Faculty of Health, Social Care and Education, Kingston University and St George’s, University of London, 6th Floor Hunter Wing, St George’s, University of London, London SW17 0RE, United Kingdom; b Bournemouth University, Faculty of Health and Social Sciences, Royal London House, Lansdowne Campus, Bournemouth University, Bournemouth BH1 3LT, United Kingdom

**Keywords:** Caregivers, Carers, Older people, Experience, Qualitative, Focus groups

## Abstract

**Background and aims:**

Unpaid family carers, or caregivers as they are also known, often play a vital role in supporting others with illness or disability living in the community. Overall numbers of carers are growing but numbers of older carers are increasing particularly rapidly as populations age worldwide. However, little research has focused on this important older group. This qualitative study therefore investigated older carers’ experiences and their perceptions of their role.

**Methods:**

Five digitally recorded focus groups with carers from Greater London were undertaken. Recordings were transcribed and analysed thematically.

**Findings:**

Forty-four carers aged 70–87 years participated. Most were female and two-thirds were spouses or partners. Overall, the carers thought their experiences were similar to those of younger adult carers and included both satisfying and challenging facets. However, they thought that some of the more negative aspects of the role were more difficult for older carers. Their own declining physical and emotional health and strength were seen as making it harder to access support and maintain social contacts. Loneliness both outside and within relationships featured prominently and was perceived as especially significant for housebound carers and when caring for someone with dementia. Many of these older carers also worried about the future when they might no longer be able to be a carer due to their own ill-health or death.

**Conclusions:**

Older carers find their role challenging and future investigations should focus on identifying means of reducing their isolation and supporting them with planning for the future.

## Highlights

• Older carers’ experiences are not dissimilar to those of other adult carers.
• These experiences may be particularly challenging due to their age.
• Loneliness both outside and within relationships is common.
• Older carers worry about the future when they cannot care.
• Older carers often fail to ask for support.

## 1 Introduction

Worldwide populations are ageing and people are living longer with long-term health conditions. In parallel, numbers of unpaid, informal, often family carers are increasing [1]. For example, in the United Kingdom (UK), there are approximately 6.5 million adult carers and this is projected to increase to 9 million by 2037. Within this overall figure, numbers of older carers (65+ years) are increasing more rapidly than other age groups. Currently there are approximately 1.3 million older carers and whilst total carer numbers have risen by 11% since 2001, numbers of older carers rose by 35% over the same period [2]. The proportion of the oldest carers, aged 85+, is growing particularly fast and has increased by 128% over the last decade [3].

Being a carer is recognised as a mixture of satisfactions and challenges [4] with some carers reporting long-term physical and emotional health problems [5]. Carers may themselves therefore benefit from support. For older carers, the need for support may be even greater as they often care for longer hours, are often co-resident and provide more intensive and intimate care [6].

Many older carers support spouses or partners, frequently of a similar age. Spousal caregiving in later life is expected to increase [7] but numbers of long-term older parent carers supporting adult children with physical and learning disabilities are also growing. However, each of these groups are likely to have slightly different experiences and concerns. For example, caring for someone with dementia is often regarded as one of the most challenging caring roles [8] whilst older carers of adult children may have greater concerns about when they can no longer care [[9], [10], [11]].

Despite their growing significance, older carers are relatively neglected in research [12,13] and little is known specifically about this group. A review of quantitative literature focusing on older carers’ quality of life [14] concluded that older people are increasingly supporting people living with dementia and greater carer age is associated with poorer perceived quality of life. A recent systematic review of both quantitative and qualitative literature relating to the experiences of carers aged 75+ years [12] reported that quantitative studies generally emphasised the difficulties of caring whilst the qualitative studies were more positive emphasising the rewards of caring and carers’ active responses. Some studies suggested caring might be less challenging for older carers and highlighted the normality of caring as part of family relationships. The authors concluded that although older carers are often included in research studies, they are seldom investigated as a specific group and few studies directly compare them with younger carers, making it difficult to know if their experiences are similar or different to other age groups. More research focusing specifically on this important group was recommended [12].

Therefore, the aim of the current study was to explore the experiences of older carers and to understand, from their perspectives, whether their experiences were similar or different to those of younger adult carers.

## 2 Methods

While individual interviews have advantages, focus groups were selected for data collection since they allow exploration amongst peers of their experiences [15] and encourage participants to explore what is important to them in their own words [16].

Two third-sector carer organisations, both based in Greater London, UK, recruited participants. To take part, carers had to be aged over 70 years. This cut-off was selected because many people are delaying retirement to after 65 years. They also had to be currently or recently caring (last two years). Sampling was purposive with the intention of recruiting a diverse range of older carers in terms of their gender, ethnic background, caring situations and care recipients’ health conditions.

Recruiters contacted carers fitting the inclusion criteria and described the project aims and methods. Those expressing an interest in participating were invited to attend a focus group. Before starting, the researchers reminded carers of the project aims, emphasised they were under no obligation to participate and that they could leave the group at any time without offering an explanation. Following written consent, carers completed a brief demographic information form which included, for example, relationships with the care recipient (e.g. spouse, adult child) and care recipients’ health conditions. Groups were facilitated by researchers experienced in moderating discussions with carers and older people.

To initiate conversation, carers were provided with a large selection of picture cards which included, for example, scenery, animals and some famous paintings. They were asked to select one or more cards fitting their caring experiences and then to explain individually why they had chosen the card and how it related to their caring experiences. Following this, the facilitator used a short topic guide to encourage participants to discuss their caring experiences. There were four main topics, what it is like being a carer in general, whether they thought their experiences were similar or different to those of younger adult carers and what, if anything they found challenging and satisfying about being a carer.

Analysis was thematic [17] and was ongoing during the study allowing focus group facilitators to incorporate their reflections of earlier findings in later groups. Using open coding, two members of the research team independently coded three early transcripts, reading and re-reading them ensuring familiarity with the data. The researchers then met and discussed their initial coding and a preliminary coding framework was developed. This process was repeated and the data categories identified were finalised and grouped together reducing their number. Following development of the final framework, later transcripts were coded and earlier ones recoded.

The project was overseen throughout by an advisory group which included carers, researchers and service providers and ethical approval was gained from the Faculty Research Ethics Committee.

## 3 Findings

### 3.1 Carer participant demographics

Forty-four carers took part in five focus groups with between six and 12 participants in each group. Groups lasted from 1.5 to two hours and were hosted in the recruiting voluntary sector organisations. Most carers were White British (82%), had English as a first language and were female (82%). Ages ranged from 70 to 87 years (average 76). Participants had been caring for nearly 20 years on average. The majority (86%) were currently caring and approximately two-thirds were caring for a spouse or partner (64%) and most of the remainder were caring for adult children (32%). A quarter (25%) were caring for a family member with dementia and the same proportion were caring for someone with mental illness. Full details are provided in Table 1.

**Table 1:** Carer characteristics.

N = 44	
Age	
Mean	76 years
Median	77 years
Range	70 - 87 years

Caring duration	
Mean	19.7 years
Median	14 years
Range	2 months – 57 years
Gender	

Female	38 (86%)
Male	6 (14%)

Ethnicity	
White British	36 (82%)
Mixed	2 (5%)
Indian	2 (5%)
Other	4 (8%)

Caring status	
Current	38 (86%)
Former	6 (14%)

Caring relationships	
Spouse/partner	28 (64%)
Adult child	14 (32%)
Parents	2 (5%)
Dual carers	4 (10%)

Care recipients’ health conditions	
Dementia	11 (25%)
Mental illness	11 (25%)
Learning/developmental disability	5 (11%)
Physical health problem (e.g. lack of mobility/falls)	3 (7%)
Stroke	3 (7%)
Parkinson’s disease	3 (7%)
Arthritis	2 (5%)
COPD	2 (5%)
No diagnosed illness/disability	2 (5%)
Other	1 (3%)

NB: Where %s do not add up to 100, this is due to rounding.

#### 3.1.1 Themes

Carers emphasised their belief that their positive attitude was central and they generally agreed that many of the experiences of older and younger adult carers are very similar, although some were perceived as more acute or more challenging for older carers. To highlight this, the findings are grouped into three sections with associated themes. Section 1 describes the perceived impact of carers’ positive attitudes. Section 2 highlights themes perceived as common to adult carers in general and Section 3 describes aspects of caring seen as particularly relevant or more difficult for older carers.

### 3.2 Section 1: attitudes to caring

#### 3.2.1 Positive outlook

Carers clearly believed that their outlook and attitude to caring and love for the care recipients had a major impact on their experiences. Many participants came across as consciously making an effort to be positive about their situations. They did not suggest that they were different to younger carers in this respect but appeared to regard it as central to being a carer. The following is a typical comment.

*‘Life is wonderful, life is what you make it. That’s the way I look at it anyway.’*

Carers explained how they had needed to accept and ignore some changes in care recipients’ behaviour.

*‘You just feel like you’re nagging the whole time, so I tend to put blinkers on now and think, “It’s okay. He might not have washed for a couple of days but it doesn’t matter.”’*

#### 3.2.2 Love and acceptance

This positivity and love for the care recipient often appeared to make it possible for carers to continue caring, despite the challenges.

*‘I look at M sometimes and I’m overwhelmed with love and I just think, “What has happened to this man?” but then two minutes later when I say, “Darling, I’ve got to do your insulin,” and he’s screaming at me, “Ooh, I hate you,” … I am always on this yoyo, going from love to hate.’*

Part of accepting their caring role seemed to be seeing it as a natural part of family life rooted in love and integral to their long-term relationships, whether as parents or spouses.

*‘I would say it’s part of the natural, that’s the mother being with the daughter, or the wife being with the husband, the natural caring relationship is the love you have.’*

### 3.3 Section 2: older carers’ experiences in common with adult carers in general: challenges and satisfactions

Carers generally felt that many aspects of caring were similar irrespective of age. This carer described a very common sentiment.

*‘I think they must have a lot in common because … the caring role is the same, it’s just perhaps… we’re older so up to a point it’s more physically demanding and tiring.’*

It was also clear from carers’ discussions that caring was a mixture of challenges and satisfactions and that their experiences changed continuously. A remark by a carer aged 80+ caring for her daughter typified this.

*‘My daughter has brought me a lot of worry and a lot of joy. You’re never free, but I’m happy with life, and I’ve got a happy family life. So, I just think she is a blessing because she’s made me a nicer person.’*

Participants emphasised repeatedly that all adult carers are different – whether younger or older and that caring is neither always challenging nor satisfying. These two overarching aspects of their experiences – the challenges and satisfactions of caring are described separately. Challenges of caring are described first.

#### 3.3.1 Loss

Carers often explicitly or implicitly described negative changes in their lives after becoming carers. The theme of loss incorporates many of these changes. It includes losses of freedom and leisure activities, reduced choices and financial losses.

*‘I was constantly looking for green shoots or light at the end of the tunnel that never came. I also felt like a caged bird and towards the end like a bird whose wings were clipped.’*

*‘… it’s that uncertainty, not being able to do the things you want to do. I love going to the theatre, but theatre tickets are expensive and I didn’t know if on the day she was going to be well or not, so I wasn’t booking tickets.’*

Spousal carers especially often thought fondly about their former life as a couple and being able to go out together. They also often missed their life outside caring and some felt they may never regain this.

*‘…a loss of the way of life and like walking, that’s what we did all the time, we walked everywhere together and lovely walks, either in London or, you know, in the countryside or whatever.’*

There were also social and emotional losses such as changed or lost relationships with care recipients, often with loss of companionship and intimacy. This was particularly relevant for carers of people with cognitive and communication difficulties such as dementia or after stroke.

*‘… my husband had a stroke 11 years ago and, in my opinion, he died then.’*

#### 3.3.2 Loneliness and isolation

In response to being asked how to describe being an older carer, this participant simply said:

*“Lonely’ in one word.’*

Carers attributed their loneliness in part to diminishing social circles. They described how they no longer saw friends who frequently do not understand the care recipients’ health conditions, especially dementia or mental illness.

*‘Your so-called friends disappear, because they don’t want to have to be involved. They perceive they could be called on and so you’re left with perhaps two or three people who’ve got problems of their own.’*

Sometimes friends seemed to withdraw but some carers deliberately avoided seeing them. Often this was because they were uncomfortable inviting others to their homes because of uncertainty about the behaviour of the care recipient.

*‘I used to do lots of things before, but you do withdraw a little … I don’t invite people to the house that much, especially if I think my husband’s not very well… if he’s going manic, people don’t understand.’*

#### 3.3.3 Carers’ physical and mental health

Carers talked at length about their own physical and mental health problems and how this affected their experiences and their ability to care. They highlighted tiredness, lack of sleep and physical strain.

*‘I get very tired, very, very tired and I don’t sleep a lot in the night, worried about him and I have a lot to do … he’s afraid that I will be going, in his head he keeps on saying, “Are you going, are you going, are you leaving me, are you leaving me?”’*

Feeling they have to do everything and being in demand all the time added to their, sometimes overwhelming, tiredness.

*‘You feel totally responsible for everything all the time. You have to do everything, there are so many hats to wear.’*

#### 3.3.4 Guilt

Despite their generally positive outlook, the carers often described feeling guilty. This was common if they went out and enjoyed themselves without the care recipient or if they became angry with them.

*‘Even if you had the facility to be able to go [out] and leave them … you end up feeling guilty.’*

Carers constantly had to remind themselves of the person they cared for before their illness. This carer had been married for 65 years.

*‘… he’ll do something stupid and I’ll shout at him and then I feel terrible, because he tries all the time and every night he tells me how he loves me and how pleased he is he married me.’*

Some continuously felt guilty because, despite their best efforts, they believed they were not doing enough.

*‘You’re doing your best but you’re guilty all the time.’*

Caring for one family member had an impact on the support that could be offered to others who might need it. This too resulted in guilt.

*‘My mother wanted to live with us and stay with us and I couldn’t have her as well, because I was just, it just would have been physically impossible for me to also look after my mother and I had to make a decision that I didn’t want to make ...’*

#### 3.3.5 Accessing and using services for care recipients and carers

*‘I used to call it battling with the professionals, because it’s just ongoing.’* [General agreement].

Although health and social care services were often appreciated, they were also perceived as creating considerable work. Carers highlighted multiple tiring, frustrating, time consuming challenges of accessing and using support. There were many conversations between participants about the scarcity of useful information, poor communication between services, onerous form-filling, non-responsive services, constantly having to chase professionals and waiting for unreliable home care workers.

*‘As good as some of them* [care workers] *were, we still had all the problems of me on the phone, constantly … “Have we got a carer today?” Because nobody came, and then my husband would shout (that’s the only thing he could do), so then I’d get called up … part of me said ‘Is it, is it worth having carers, because to me it was more stressful?”’*

There was also a lot of discussion about poor services in terms of the quality of the support and professionals’ generally poor understanding of, for example dementia, adding to their stress.

*‘And she [*care worker*] says, “Well he says that he doesn’t want to shave,” I said, “No, but he has Alzheimer’s and senile dementia, he can’t really tell you, you know, he’s going to say whatever.” …. “Please, I want him shaved because he goes to the day centre, he needs to look presentable so please shave him,” you know, and she will, but it’s a bit of, you know, it’s stressful having to…’*

Some participants felt unable to complain fearing that there might be repercussions. As a result, they sometimes gave up accessing support.

*‘You just feel “Oh I can’t* [complain]. *Never mind, I’ll do it.”’*

Poor communication between services and providers and between services and carers added to carers’ challenges. A dearth of information meant they did not know what to ask for and as a result missed out on accessing services or equipment that would have made a difference to their lives.

*‘You were having to get up in a morning, get her into a wheelchair, into a wet room, she was doubly incontinent so she had to be showered every single day but we didn’t know that there were other facilities - that we could have had a hoist …’*

General practices were seen as central to carers’ experiences and could be very helpful making a huge difference to carers whether in terms of emotional support or signposting to services. However, comments about support from general practice were very mixed with some describing them as excellent and ‘*caring*’ whilst others felt dismissed. This carer had a very positive experience.

*‘Well my GPs been great … she said, “Oh, I can arrange for the blood test people to come to you,” and I can’t tell the relief of just that, that I didn’t have to get him washed, you know, because it’s a struggle. I mean he can do it, but he’s not going to do it, and it was like I wouldn’t have to do all that nagging to get him out of the house… such a relief.’*

However, this carers’ experience was very different.

*‘You go in and they say, “How are you?” and then you say, “Well I’m not coping at the moment, you know, I need… at the moment I’m throwing him out of the front door sometimes” and he says, “Oh dear” and that’s it!’*

#### 3.3.6 Sense of achievement, fulfilment and personal growth

However, carers did not only discuss the challenges of caring and often highlighted the positives. The pleasures and satisfactions derived from caring are described next. These were varied and although the dominance of these themes varied with each focus group, some carers were very clear, almost passionate about these positive aspects of caring.

Carers described how they had learnt new skills, had grown as a person or how caring had *‘opened doors’* or was a *‘journey of discovery’*. Some would not have chosen different lives.

*‘They say you grow from the difficult experiences of life and not from the good things that happen to you, and I, yeah, I’ve grown, and I’ve, well I’ve learnt coping strategies.’*

Caring gave some carers a sense of purpose and pride.

*‘I think of a tree, my branches which I support and that gives me a purpose in life and that’s the main thing I get from caring, is a purpose.’*

*‘You’re married for better or for worse, so duty does come into it and you feel a sense that you’ve achieved a sense of vocation as well, dedication, pride in what you’re doing.’*

#### 3.3.7 Caring can be enjoyable

These older carers sometimes described the happiness and enjoyment that caring had brought. One carer explained that she felt blessed to have been a carer for over five decades despite having now to care for two adult children.

*‘You sort of get content with life, because that’s all part of your life, I sort of find, being a carer is just part of my life, I don’t find it miserable… I just think but I’ve been lucky, very happily married and I enjoy my children. Okay, my children are not perfect... I know they love me, so I enjoy it.’*

Another who was caring for her daughter with mental health problems said:

*‘… there have been some nice deep and meaningful moments and sort of that reminded me that I wouldn’t have had them if we hadn’t been in this situation. So it’s not all been bad, although it does seem so at times.’*

### 3.4 Section 3: experiences of caring perceived as more specifically related to being an older carer

Carers were asked to try and identify how, as older carers, their experiences might be similar or different to those of younger adult carers. For carers who had been in the role for many years, this involved comparing earlier experiences when younger with what is was like currently. Others, newer to the role, tended to reflect on and discuss their age and perceptions of how being older might or might not influence their experiences. Those aspects identified as challenging appeared to be made more difficult with age but carers did not highlight any greater satisfaction as older carers, although some did say that prior use of services may make their lives easier by helping them to know about available support. There also appeared to be some differences between the oldest carers (late seventies and older) and those in their early seventies. These are highlighted in the themes below.

#### 3.4.1 Older carers’ fears for the future

One common concern for these carers was the future. Many worried about being unable to continue caring through illness or death, especially if they were caring for someone significantly younger than themselves such as an adult child. This concern was mentioned by many carers but seemed to be more of an issue for the oldest carers.

*‘I was thinking as you get older it’s a lot scarier, you know that you are that one step nearer the end of your journey and what exactly is going to happen after you’ve reached that end? Now it could be a stroke, it could be death, but death is almost the easy way for you but if you had a stroke…’*

This carer was concerned about her daughter’s vulnerability.

*‘… she doesn’t read people … and she’s a very caring person so she’ll help somebody if they look like they need help. So, being on her own that does worry me. And me getting older… that worries me very much, who will look after her and who will care for her?’*

One participant, caring for her husband highlighted her day-to-day concerns.

*‘I worry about if I’m not well enough to look after him - what would happen? Or is he aware that if someone is ill what to do? I mean if I had a heart attack, would he know what to do then, would he be able to call 999 or would he call a neighbour?’*

#### 3.4.2 Older carers are less likely to ask for support

Carers of all ages frequently do not seek support for their role but participants here thought older carers were even less likely to ask for help than younger carers. A range of explanations were offered including older people having a stronger sense of duty or pride making them more likely *‘to soldier on’* and less likely to expect support.

*‘I mean I was born before the war and we had a sense of duty to our parents and they don’t have that now.’*

*‘It’s also the reluctance to ask as well because you don’t feel like you’re entitled … because I can actually do it, you know, I can help him in and out of the bath, I can do it.’*

Some carers also highlighted that as they aged their lower energy levels and stamina meant they tended to give up asking for or trying to access services.

*‘She would be dead I think if it hadn’t been for us … but I haven’t bothered this time to get carers, because I’m just worn out.’*

#### 3.4.3 Older carers’ physical and mental health

Perhaps the most striking perceived difference between the experiences of older and younger carers was older carers’ generally poorer health. This was often said to be reflected in specific health conditions and lower energy levels which affected how they experienced their role. Some felt they could not continue much longer. Carers in their early seventies and those in older groups both mentioned this but many of the younger carers here talked about the future when they were less physically able, whilst the oldest carers more often referred to current health problems and fatigue.

*‘Well I’ve got to deal with getting older, you’re losing your hearing, your sight, your teeth, your hair, [*laughter*]. For example, I had a fall just before Christmas, I fell down the stairs … and it’s a lack of sleep in that situation, that’s what I really noticed, because I had in pain with my legs, I was full of tension and anxiety for my daughter… you don’t have that same energy level.’*

Others highlighted poorer mental health and strength.

*‘I find my strength is going, I don’t mean physical strength, I mean my own mental strength, you know, when you’ve got to say something* 3000 *times, it’s like having an 85 year old two year old at home. You tell a two-year-old and it does what you tell it to do, but an 85 year old doesn’t.’*

#### 3.4.4 Older carers are more likely to be lonely and socially isolated

Loneliness and isolation are recognised as common amongst carers but older carers here thought they were even more likely to be lonely and socially isolated than younger carers. This was because of decreasing social networks associated with age in general but also because of their reduced energy. Isolation was thought to be a particular problem if carers were housebound, often because of their own health or caring responsibilities. This issue was associated with age, with the oldest carers being seen as most likely to be housebound as a result of their health, the care recipients’ health, or having few surviving friends.

*‘You are very isolated as an older person, I have lost most of my friends that I used to meet up with and go out with … I can’t do anything like that because I can’t leave him.’*

Many carers described how loneliness and isolation became worse as caring progressed. Not only had their social circles diminished but loneliness within their relationships was also an issue, usually the result of changed relationships for carers of spouses with dementia.

*‘Ours is a loneliness within the relationship, I’m not lonely outside because I have care workers coming in, and Alzheimer’s, the church and still many lovely friends. No, so it’s a different loneliness, it’s just being in the house, I can’t talk to my husband, he doesn’t understand.’*

*‘We’re sitting here both of us, and sometimes I feel we’re not together at all … I have lost the person that I married, we can’t really share, he can speak and walk and everything but he doesn’t want to, he just wants to live in a silent world of quietness.’*

## 4 Discussion

The findings from this study highlight older carers’ perceptions of the similarities and differences in experiences between younger and older adult carers. As reported previously caring was described as a constantly changing mixture of satisfactions and challenges [10,12]. Our participants highlighted their love for care recipients and the importance of having positive attitudes to caring. Many of the experiences described by these older carers are very similar to those reported by younger adult carers but some of these, especially the challenging experiences, were perceived as often more difficult or worse for older carers. Findings here therefore support the suggestion that being an older carer may often be more challenging than for younger adult carers [18]. Like adult carers more generally [19], these carers described the stamina required to overcome the barriers to accessing formal support and emphasised that increasing age made this harder. Furthermore, physical caring activities which they had previously not found difficult were now more challenging. Added to this, carers’ social circles, and therefore also potential social support, diminished with age whilst their own health conditions sometimes made it harder to leave home to maintain friendships. These carers believed they may be even less likely than younger carers to request support partially because of pride but also because compared to younger generations, they were less likely to believe they were entitled to support. This pride and reluctance to seek support has also been reported by volunteers and professionals supporting older carers [20]. The fact that they had been caring for so long and their caring was rooted in long-term relationships [21] meant they were often resistant to accepting help. Seeing their role as a natural part of their love for care recipients and family relationships may have also contributed to their ambivalence about requesting outside help.

Loneliness and isolation amongst carers are frequently reported [2,20,22,23]. These issues were commonly mentioned here and often thought to be exacerbated by carers’ age, ill-health and the fact that many were caring for stigmatised, often poorly understood conditions such as dementia [24]. Many spousal older carers here were supporting partners with dementia and the symptoms and other people’s poor understanding of the condition, further limited activities outside home [24]. Our participants described negatively changed relationships [25] and distinguished between loneliness outside and within relationships, for example, when the person they were caring for was unable to communicate as a result of advanced dementia. This loneliness within relationships in particular deserves further exploration.

Carers’ advancing age, meant many were concerned about having plans in place for looking after their loved ones if they were unable to care for them because of ill-health or death. Volunteers and professionals working with older carers also highlight this [20] but suggested that carers sometimes avoided discussing this. These different perspectives have implications for supporting carers and deserves further investigation.

The study did not specifically aim to compare carers from different sub-groups of older carers – for example, males and females or those caring for a long time compared to those new to the role. However, there did appear to be some differences between the very oldest carers (late seventies and older) and those in their early seventies. Perhaps unsurprisingly, the oldest carers appeared to be more often concerned about their own declining health and the care of their loved ones when they die or are unable to care. Greater carer age also appeared to be associated with increased chances of being housebound and socially isolated. Although we did not detect differences in other respects, it is impossible to say whether there were differences due to a fairly homogenous carer sample. Similarities and differences between these sub-groups deserves further investigation in future studies.

This study had a number of strengths including the in-depth discussion amongst a relatively large number of older participants who were caring for people with a wide range of health conditions. Many had cared for several decades giving a detailed picture of their experiences over time. However, there are limitations. For example, carers had to be physically able to attend the focus groups, meaning that, for example, housebound carers were unable to participate. Additionally, all participants came from greater London, UK and despite active efforts to include a diverse range of carers, they were largely female and mostly described themselves as White British. This meant it was not always possible to compare different groups of carers, for example male and female carers or to compare different ethnic groups. Furthermore, as they were recruited via carers organisations, they had been in contact with some support services and therefore their experiences may not all reflect the experiences of those not receiving any support. Finally, the carers’ accounts were also often retrospective comparisons of caring when younger and may have been influenced by the biases inherent in such descriptions.

Future research should focus on longitudinal studies over extended periods to monitor carers’ experiences as they age and data collection methods could include, for example, telephone interviews to allow housebound carers to participate. Carers are as diverse as those they care for and to help understand these different situations, future studies might focus on comparisons between specific groups of older carers’, for example, those supporting adult children with learning difficulties or mental health problems. It is also possible that older carers living in rural and urban environments or carers from minority ethnic groups may have very different experiences. Worries about the future when they can no longer care through death or illness were common and included spousal carers caring for someone of a similar age. Further investigation of all these topics would be valuable.

## 5 Conclusions

Being an older carer is both challenging and satisfying and services need to take into account older carers’ distinctive characteristics. For example, more socially orientated activities that carers and care recipients can attend together such as dementia cafés [26] and ‘Singing for the Brain’ [27] allow both carers and care recipients to meet others to gain social and peer support but also mean that the carer need not feel guilty for leaving the care recipient at home while they enjoy themselves. They also offer environments where others understand health conditions such as dementia and where unusual behaviour is accepted. Voluntary sector services which offer carers peer support and mentoring either at home or via the telephone may be particularly beneficial for housebound older carers. In addition, these older carers are frequently concerned about their failing stamina and health and worry about what the future holds for the person they care for. Interventions to support them with this difficult issue are needed but this is a sensitive and complex topic and may need involvement of multiple services, for example, older people’s services, learning disability services and the voluntary sector.

## Contributors

Nan Greenwood conceived of and led the study, contributed to data collection and analysis, drafted the paper and contributed to revision of the manuscript.

Carole Pound contributed to data collection and revision of the manuscript.

Sally Brearley contributed to data collection and revision of the manuscript.

Raymond Smith contributed to data collection and analysis, and revision of the manuscript.

All authors saw and approved the final version of the paper.

## Conflict of interest

The authors declare that they have no conflicts of interest.

## Funding

The Wellcome Trust funded the study [209343/Z/17/Z]. The Trust was not involved in the study in other respects.

## Ethical approval

Ethical approval was granted by the Faculty of Health, Social Care and Education, Kingston University research ethics committee (Ref FREC 2017-11-004). Participants consented to anonymised quotes being used in dissemination.

## Provenance and peer review

This article has undergone peer review.

## Research data (data sharing and collaboration)

There are no linked research data sets for this paper. The authors do not have permission to share data.
